# Metformin Use and Development of Esophageal Squamous Cell Carcinoma

**DOI:** 10.1001/jamanetworkopen.2026.2027

**Published:** 2026-03-16

**Authors:** Shao-Hua Xie, Giola Santoni, Helgi Birgisson, My von Euler-Chelpin, Joonas H. Kauppila, Eivind Ness-Jensen, Jesper Lagergren

**Affiliations:** 1 Upper Gastrointestinal Surgery, Department of Molecular Medicine and Surgery, Karolinska Institutet, and Karolinska University Hospital, Stockholm, Sweden; 2 Icelandic Cancer Registry, Icelandic Cancer Society, Reykjavik, Iceland; 3 Department of Public Health, University of Copenhagen, Copenhagen, Denmark; 4 Surgery Research Unit, Oulu University Hospital and University of Oulu, Oulu, Finland; 5 HUNT Research Centre, NTNU, Levanger, Norway; 6 Department of Medicine, Levanger Hospital, Nord-Trøndelag Hospital Trust, Levanger, Norway; 7 School of Cancer and Pharmaceutical Sciences, King’s College London, and Guy’s and St Thomas’ National Health Service Foundation Trust, London, United Kingdom

## Abstract

**Importance:**

Esophageal squamous cell carcinoma (ESCC) carries a poor prognosis, stressing the need for preventive measures. A decreased risk of ESCC among metformin users has been suggested, but evidence is limited.

**Objective:**

To assess whether metformin use, given its potential anticancer properties, is associated with risk of ESCC.

**Design, Setting, and Participants:**

This population-based case-control study set between 1994 and 2023 looked at data from all 5 Nordic countries (ie, Denmark, Finland, Iceland, Norway, and Sweden). Participants were patients newly diagnosed with ESCC during the study period, and each was compared with 10 times as many control participants randomly selected from the general population (matched by age, sex, calendar year, and country).

**Exposures:**

Use of metformin vs nonuse.

**Main Outcomes and Measures:**

Conditional logistic regression provided odds ratios (OR) with 95% CIs for the association between metformin use and the development of ESCC. In addition to the matching, the ORs were adjusted for tobacco smoking, alcohol overconsumption, use of nonsteroidal anti-inflammatory drugs or aspirin, and use of statins. A dose-response analysis was conducted among participants with at least 5 years of observation time, based on the defined daily dose during the 5-year period.

**Results:**

This study included 13 050 case patients with ESCC (8030 men [61.5%] and 5020 women [38.5%], diagnosed at a median age of 70 years [IQR, 62-77 years]), and 130 500 age- and sex-matched control participants. Metformin use was associated with a 36% lower odds of ESCC compared with nonuse (OR, 0.64; 95% CI, 0.59-0.69). The odds were especially lower in participants with a higher dosage of metformin (>1278 defined daily dose in 5 years: OR, 0.52; 95% CI, 0.44-0.61).

**Conclusions and Relevance:**

In this case-control study, metformin use was associated with substantially lower odds of ESCC. This finding should prompt investigations of metformin as a preventive option in high-risk individuals and as a potential future therapeutic agent for ESCC.

## Key Points

**Question:**

Dose use of metformin reduce the risk of developing esophageal squamous cell carcinoma (ESCC)?

**Findings:**

In this case-control study with 13 050 patients with ESCC and 130 500 control participants, metformin use was associated with 36% lower odds of ESCC. This was more profound among high-dosage users.

**Meaning:**

These findings suggest metformin use may have preventive potential against ESCC, which warrants further evaluation.

## Teaser

This case-control study investigates potential associations between the use of metformin and risk of esophageal squamous cell carcinoma in Denmark, Finland, Iceland, Norway, and Sweden.

## Introduction

Esophageal cancer is the seventh leading cause of cancer-related deaths globally, with esophageal squamous cell carcinoma (ESCC) as the predominant type, accounting for over 80% of all cases.^1,2^ The global incidence of ESCC has declined over time, but a rising incidence has been noted in women in several countries.^3^ Tobacco smoking and alcohol overconsumption are the major established risk factors for ESCC in Western populations, while dietary risk factors, such as low intake of fresh fruit and vegetables, may also play a role. Some studies have suggested that long-term use of nonsteroidal anti-inflammatory drugs (NSAIDs) or aspirin and statins seem to decrease the risk of ESCC.^1,4^ Given the poor prognosis of ESCC, with an overall 5-year survival rate of less than 20%,^5^ there is a great need for effective preventive measures.

Metformin is a first-line medication in the treatment of diabetes and may also be prescribed for other insulin-resistant conditions, such as polycystic ovary syndrome, with a favorable safety profile and minimal toxic effects. Metformin has potential anticarcinogenic properties, and its use may reduce the risk of cancer overall and especially gastric, colorectal, urologic, and hematologic cancers.^2,6,7,8,9^ However, evidence regarding the association between metformin use and the risk of ESCC has been largely absent. Our previous Swedish cohort study indicated that metformin use was associated with a 32% reduced risk of ESCC.^10^ This finding has not been examined in larger studies with longer follow-up, which would allow subgroup analyses and evaluation of dose-response patterns. To help clarify the hypothesis that metformin use is associated with a decreased risk of developing ESCC, we conducted a population-based case-control study across the 5 Nordic countries, utilizing high-quality registry data to allow for a large study with long and complete observation period, enabling sufficient statistical power to assess dose-response associations and differences in associations between subgroups.

## Methods

### Study Design and Participants

This population-based case-control study was conducted across the 5 Nordic countries (Denmark, Finland, Iceland, Norway, and Sweden) between 1994 and 2023, as part of the Nordic Gastric and Esophageal Tumor Study (NordGETS). The NordGETS includes all patients with a newly diagnosed gastric or esophageal cancer during the study period, who were identified from the national cancer registries in these countries, and 10 times as many control participants randomly selected from national population registries.

This study included all patients in NordGETS with a newly diagnosed ESCC, according to tumor topography and histology codes listed in eTable 1 in Supplement 1. The date of ESCC diagnosis served as the index date for each case. For each case, 10 control participants without a history of esophageal cancer and alive on the case’s index date were randomly selected and matched by age, sex, calendar year, and country. The overall study period from 1994 to 2023 allowed an observation (or latency) period for up to 26 years. However, the start of the observation varied by country depending on when the nationwide prescribed drug registries started, ie, in 1994 in Denmark and Finland, 2002 in Iceland, 2004 in Norway, and 2005 in Sweden.^11,12,13^

Approvals were obtained from all relevant ethical review boards, governmental agencies and data inspectorates in the participating countries. Informed consent was waived for registry-based research in the Nordic countries. This study followed the Strengthening the Reporting of Observational Studies in Epidemiology (STROBE) reporting guideline for case-control studies.^14^

### Data Sources

The NordGETS integrates data from multiple nationwide health data registries available in all Nordic countries. By means of the unique personal identity number assigned to each resident, individuals’ data from various registries can be linked with high accuracy. The registries that provided data for the study were:

- Cancer registries, which have virtually complete coverage of cancer diagnoses with high-quality data on the topography, histology, and the diagnosis date.^15,16,17,18,19^ These registries were used to identify the cases of ESCC.
- Prescribed drug registries, which record all prescribed and dispensed medications from all pharmacies, including drug name, Anatomical Therapeutic Chemical (ATC) code, dispense date, and quantity.^11,12^ They enabled accurate longitudinal assessment of metformin use and other medication exposures.
- Patient registries, including inpatient and outpatient registries, which capture all diagnoses.^20,21,22,23^ These data were used to ascertain diagnoses related to the risk factors adjacent to tobacco smoking and alcohol overconsumption, among other comorbidities, for confounder adjustment.
- Cause of death registries, which have 100% complete coverage regarding date of death.^24,25,26,27^ The data were used to establish vital status and ensure that control participants were alive on the index date.

### Exposure

The use of metformin was ascertained from the national prescribed drug registries using corresponding ATC codes (eTable 2 in Supplement 1). For each participant, the complete history of metformin dispensations was retrieved from the start of the prescription registry until the index date. Individuals with at least 2 dispensations of metformin before the index date were classified as exposed. Two dispensations were required to exclude short-term use.

### Covariates

Eight variables were included as covariates: (1) age as of the index date; (2) sex; (3) calendar year of index date; (4) country; (5) tobacco smoking (smoking-related diagnoses before index date); (6) alcohol overconsumption (alcohol-related diagnoses before index date); (7) use of NSAIDs or aspirin before index date; and (8) use of statins before index date. The specific diagnoses and ATC codes used in each country to define these covariates are provided in eTables 2 to 4 in Supplement 1.

### Statistical Analysis

Conditional logistic regression was used to assess the association between metformin use and the development of ESCC, providing odds ratios (ORs) with 95% CIs calculated using Wald statistics based on model-derived SEs. In a crude model, the OR estimates were controlled for the 4 matching covariates by incorporating the group variable for the matched pairs as strata. In a full model, we additionally adjusted for the covariates tobacco smoking (yes or no), alcohol overconsumption (yes or no), use of NSAIDs or aspirin (yes or no), and use of statins (yes or no). Information on tobacco smoking and alcohol overconsumption was missing for 3047 participants in Norway (2.1% of all participants) because the Norwegian Patient Register was established later than the Norwegian Prescribed Drug Registry, and these individuals were excluded from the fully adjusted model. No other missing data were identified.

Among participants with at least 5 years of observation time (ie, index date minus registry start date ≥5 years), we assessed the average defined daily doses (DDD) of metformin during the 5 years preceding the index date. A dose-response analysis was performed among these participants, in which metformin users were categorized into 3 approximately equal-sized groups (tertiles), ie, low (≤460 DDD), medium (461-1278), and high (>1275 DDD), with nonusers as the reference group. In an additional analysis among metformin users, the number of DDD per week was modeled as a continuous variable.

We examined potential effect modification by sex (male and female), age (≤65, 66-74, and ≥75 years), calendar year (1994-2000, 2001-2010, and 2011-2023), tobacco smoking (yes and no), alcohol overconsumption (yes and no), use of NSAIDs or aspirin (yes and no), and use of statins (yes and no). For this purpose, an interaction term between the exposure and the covariate was added to the model, and stratum-specific associations were estimated. Each potential effect modifier was assessed in a separate model. Age and calendar year were categorized using predetermined cutoff points, and all effect modifiers were entered as indicator variables, with the first listed category as the reference. To account for multiple testing, *q* values were calculated using the Benjamini-Hochberg method.

To assess potential detection bias, we performed a sensitivity analysis that excluded metformin use and the covariates within 6 months before the index date. All statistical analyses were carried out using the statistical software Stata version 19 (StataCorp). A senior biostatistician (G.S.) conducted all data management and statistical analysis according to a predefined protocol. All statistical analyses were 2-sided and *P* < .05 was considered statistically significant.

## Results

### Participants

This study included 13 050 patients with ESCC (8030 [61.5%] men and 5020 [38.5%] women) and 130 500 control participants. The median age on the index date was 70 years (IQR, 62-77 years). Compared with control participants, more patients with ESCC had smoking– and alcohol-related diagnoses before the index date, whereas no differences were observed between patients with ESCC and control participants in the use of NSAIDs or aspirin or the use of statins (Table 1).

**Table 1.** Characteristics of Cases of Esophageal Squamous Cell Carcinoma and Control Participants

Characteristics	Participants, No. (%)	
	Case patients (n = 13 050)	Control participants (n = 130 500)
Age, y		
18-65	4774 (36.6)	47 279 (36.2)
66-74	4223 (32.4)	41 984 (32.2)
≥75	4053 (31.1)	41 237 (31.6)
Median (IQR)	70 (62-77)	70 (62-77)
Sex		
Male	8030 (61.5)	80 300 (61.5)
Female	5020 (38.5)	50 200 (38.5)
Calendar year		
1995-2000	1745 (13.4)	17 450 (13.4)
2001-2010	4621 (35.4)	46 210 (35.4)
2011-2023	6684 (51.2)	66 840 (51.2)
Country		
Denmark	5061 (38.8)	50 610 (38.8)
Finland	3096 (23.7)	30 960 (23.7)
Iceland	121 (0.9)	1210 (0.9)
Norway	1311 (10.1)	13 110 (10.1)
Sweden	3461 (26.5)	34 610 (26.5)
Tobacco smoking^a^	1567 (12.0)	6739 (5.2)
Alcohol overconsumption^a^	2311 (17.7)	4024 (3.1)
Use of nonsteroidal anti-inflammatory drugs or aspirin	9096 (69.7)	92 110 (70.6)
Use of statins	3711 (28.4)	38 436 (29.5)

^a^ Missing for 3047 participants in Norway (2.1% of all participants).

### Metformin Use and Risk of ESCC

A total of 725 patients with ESCC (5.6%) and 10 448 control participants (8.0%) were metformin users. Metformin use was associated with an overall lower odds of ESCC (adjusted OR (AOR), 0.64; 95% CI, 0.59-0.69) (Table 2). Compared with nonusers, the AORs were 0.67 (95% CI, 0.59-0.77) for low-dosage users, 0.66 (95% CI, 0.57-0.76) for medium-dosage users, and 0.52 (95% CI, 0.44-0.61) for high-dosage users of metformin (Table 2). The dose-response analysis treating the exposure as a continuous variable among metformin users showed an AOR of 0.95 (95% CI, 0.90 to <1.00) for each increase in DDD per week (Table 2). The overall association remained virtually unchanged in the sensitivity analysis that excluded metformin use and covariates within 6 months before the index date (AOR, 0.65; 95% CI, 0.60-0.71).

**Table 2.** Metformin Use and the Risk of Esophageal Squamous Cell Carcinoma

Exposure	No.		OR (95% CI)	
	Case patients	Control participants	Crude^a^	Adjusted^b^
Metformin use				
No	12 325	120 052	1 [Reference]	1 [Reference]
Yes	725	10 448	0.67 (0.62-0.72)	0.64 (0.59-0.69)
Total defined daily doses of metformin use in 5 y^c^				
None	9667	93 671	1 [Reference]	1 [Reference]
≤460	241	3120	0.74 (0.65-0.85)	0.67 (0.59-0.77)
461-1275	230	3174	0.70 (0.61-0.80)	0.66 (0.57-0.76)
>1275	172	3135	0.52 (0.45-0.61)	0.52 (0.44-0.61)
Defined daily doses per week^d^	NA	NA	0.93 (0.89-0.98)	0.95 (0.90 to <1.00)

Abbreviations: NA, not applicable; OR, odds ratio.

^a^ Matched for age, sex, calendar year, and country.

^b^ Matched for age, sex, calendar year, and country and adjusted for tobacco smoking, alcohol overconsumption, use of nonsteroidal anti-inflammatory drugs or aspirin, and use of statins.

^c^ Limited to participants with at least 5 years of observation period for medication use prior to index date.

^d^ Limited to metformin users with at least 5 years of observation period for medication use prior to index date.

Metformin use was associated with lower odds of ESCC in all subgroups of sex, age, calendar year, tobacco smoking status, alcohol overconsumption status, use of NSAIDs or aspirin, and use of statins (Table 3). The OR estimates were similar across these subgroups, except for stronger associations among individuals aged 66 to 74 years (OR, 0.56; 95% CI, 0.49-0.64) and among those with smoking-related diagnoses (OR, 0.43; 95% CI, 0.34-0.54) (Table 3).

**Table 3.** Metformin Use and the Risk of Esophageal Squamous Cell Carcinoma, Subgroup Analyses^a^

Subgroup	No.		OR (95% CI)^b^	*P* value for interaction^c^	*q* value^d^
	Case patients	Control participants			
Age, y					
18-65	4774	47 279	0.71 (0.60-0.83)	.05	.27
66-74	4223	41 984	0.56 (0.49-0.64)		
≥75	4053	41 237	0.68 (0.60-0.78)		
Sex					
Male	8030	80 300	0.67 (0.61-0.74)	.10	.41
Female	5020	50 200	0.58 (0.50-0.67)		
Calendar period					
1995-2000	1745	17 450	0.68 (0.43-1.08)	.96	.96
2001-2010	4621	46 210	0.64 (0.54-0.76)		
2011-2023	6684	66 840	0.64 (0.58-0.70)		
Tobacco smoking					
No	1567	6739	0.68 (0.62-0.74)	<.001	.001
Yes	11 483	123 761	0.43 (0.34-0.54)		
Alcohol overconsumption					
No	2311	4024	0.66 (0.60-0.72)	.09	0.41
Yes	10 739	126 476	0.55 (0.45-0.66)		
Use of nonsteroidal anti-inflammatory drugs or aspirin					
No	9096	92 110	0.71 (0.57-0.87)	.32	0.94
Yes	3954	38 390	0.63 (0.57-0.69)		
Use of statins					
No	3711	38 436	0.63 (0.54-0.73)	.78	0.96
Yes	9339	92 064	0.64 (0.58-0.71)		

^a^ The reference category in all analyses is nonusers of metformin.

^b^ Matched for age, sex, calendar year, and country, and adjusted for tobacco smoking, alcohol overconsumption, use of nonsteroidal anti-inflammatory drugs or aspirin, and use of statins, except for the assessed variable.

^c^ Assessed by adding an interaction term between the exposure metformin use and the covariate in the model.

^d^ Computed using Benjamini-Hochberg method.

## Discussion

This case-control study suggests substantially lower odds of ESCC associated with metformin use, which was consistent across subgroups, and in particular among high-dosage users. A few previous studies have investigated the association between metformin use and the risk of esophageal cancer,^29,30,31,32,33,34^ but the majority of them did not distinguish between the histological subtypes, which have markedly different etiology. To our knowledge, only 1 Swedish population-based cohort study (from our research group) has specifically examined the association of metformin use with the risk of ESCC, and it found a 32% reduced risk of ESCC in metformin users compared with nonusers,^10^ which is in line with the results of this study. A retrospective analysis of the use of multiple medications among patients with and without ESCC across 9 hospitals in Japan found a 58% reduced odds of ESCC associated with metformin use.^35^ The inverse association between metformin use and the risk of ESCC is unlikely to be attributable to diabetes itself, as diabetes is not expected to decrease such risk, and the existing evidence does not support any association between diabetes and the risk of ESCC.^36^ Taken together with the results of the present study, the evidence supporting a protective role of metformin use against the development of ESCC seems consistent.

The anticancer properties of metformin have been investigated extensively in experimental research, and some studies have specifically addressed ESCC. Metformin is thought to exert its effects primarily through activation of AMP-activated protein kinase and suppression of the mechanistic target of rapamycin inhibitor-signaling pathway, which leads to inhibition of cell proliferation, induction of apoptosis, and regulation of autophagy.^37,38,39,40,41^ Additional mechanisms include improvement of insulin resistance,^42,43^ modulation of inflammatory and immune responses,^44,45,46^ and alterations in cellular metabolism, particularly mitochondrial function and oxidative stress.^47,48,49,50^ In addition, metformin has been reported to influence cancer stem cell activity and enhance sensitivity to chemotherapy and radiotherapy.^51,52,53,54^ Despite these observations, additional studies are needed to clarify the specific biological pathways underlying the potential protective role of metformin in the development of ESCC.

From a clinical perspective, the observed association between metformin use and a significantly decreased risk of ESCC suggests a possible role of this drug in cancer prevention and treatment. The findings provide a rationale for exploring metformin as a preventive option in high-risk individuals, for example those with precancerous lesions. The results also suggest a value of examining whether metformin medication after curative treatment for ESCC may improve survival. The majority of patients who undergo curative treatment for ESCC experience tumor recurrence leading to death within a few years after the treatment. Adjuvant treatment with metformin could potentially reduce this risk. The value of examining metformin as a preventive or therapeutic target in selected individuals, even in those without diabetes, is supported by the good safety profile, wide availability, and low cost of the treatment. However, current evidence remains limited, and before metformin may be recommended for these purposes, additional observational research followed by randomized clinical trials are needed.

### Strengths and Limitations

Strengths of this study include the population-based design, large size, long observation period, and the use of prospectively collected registry data of high quality and completeness across the 5 Nordic countries. The identification of all eligible ESCC cases and control participants randomly selected from the general population minimized selection bias. The exposure information was prospectively and continuously collected, counteracting differential misclassification. The use of a case-control design avoided immortal time bias, which has been indicated in previous cohort studies on the associations between metformin use and cancer risk.^28^ Confounding was counteracted by the matching or adjustment for the main risk factors for ESCC. In addition, we assessed possible detection bias in a sensitivity analysis. Together, these efforts added to the validity of the results.

This study also has several limitations. First, the use of proxy diagnoses for assessing confounding by tobacco smoking and alcohol overconsumption likely captured only heavy exposure. On the other hand, these measures are more objectively assessed compared with self-reporting. Second, data on socioeconomic status, race and ethnicity, dietary risk factors, and inherited susceptibility were not available. However, the strong risk reduction associated with metformin use in this study should not be explained by these factors, because they are unlikely to be strongly associated with metformin exposure, and also, their associations with ESCC risk are often modest. Third, the exposure assessment was based on drug dispensation records rather than actual use, and adherence could not be confirmed. However, requiring at least 2 dispensations to define exposure should have reduced any exposure misclassification. Additionally, although the study included all cases of ESCC in 5 Western countries, the findings may not be directly generalized to populations with higher ESCC incidence and different etiological profiles, such as in Asian countries.

## Conclusions

In summary, this large, multinational, and population-based case-control study across the 5 Nordic countries found that metformin use was associated with a lower odds of ESCC. The findings support research evaluating metformin as a possible preventive option in individuals with high risk of ESCC and as a potential adjuvant therapeutic agent among patients who have undergone curatively intended treatment for ESCC.

## Notes

Supplement 1. eTable 1. Diagnosis codes for identifying esophageal squamous cell carcinoma eTable 2. Anatomical Therapeutic Chemical (ATC) codes for exposure and covariates eTable 3. International Classification of Diseases (ICD) codes for smoking-related diagnoses eTable 4. International Classification of Diseases (ICD) codes for alcohol-related diagnoses

Supplement 2. Data sharing statement
