# Supplementary material for: Metformin Use and Development of Esophageal Squamous Cell Carcinoma
Source: JAMA Netw Open. 2026 Mar 16;9(3):e262027. doi: 10.1001/jamanetworkopen.2026.2027 (PMC12993697; doi:10.1001/jamanetworkopen.2026.2027)
Supplement: Supplement 1. — eTable 1. Diagnosis codes for identifying esophageal squamous cell carcinoma eTable 2. Anatomical Therapeutic Chemical (ATC) codes for exposure and covariates eTable 3. International Classification of Diseases (ICD) codes for smoking-related diagnoses eTable 4. International Classification of Diseases (ICD) codes for alcohol-related diagnoses [file jamanetwopen-e262027-s001.pdf]

Supplemental Online Content

Xie S, Santoni G, Birgisson H, et al. Metformin use and development of esophageal squamous cell carcinoma. *JAMA Netw Open.* 2026;9(3):e262027.
doi:10.1001/jamanetworkopen.2026.2027

eTable 1. Diagnosis codes for identifying esophageal squamous cell carcinoma

eTable 2. Anatomical Therapeutic Chemical (ATC) codes for exposure and covariates

eTable 3. International Classification of Diseases (ICD) codes for smoking-related diagnoses

eTable 4. International Classification of Diseases (ICD) codes for alcohol-related diagnoses

This supplemental material has been provided by the authors to give readers additional information about their work.

© 2026 Xie S et al. *JAMA Network Open*.

eTable 1. Diagnosis codes for identifying esophageal squamous cell carcinoma

Classifications	Codes
**Topography codes**	
International Disease Classification, 7th version (ICD-7)	150
or	
International Disease Classification, 10th version (ICD-10)	C15
**Histology codes**	
WHO/HS/CANC/24	146
or	
International Classification of Diseases for Oncology, 2nd or 3rd version (ICD-O-2 or ICD-O-3) *	8050–8078, 8083–8084

* For ICD-O-3: 5th digit = 3 or behaviour variable = 3 (5th digit = 3 or 9 in Denmark) indicating malignant tumor.

© 2026 Xie S et al. *JAMA Network Open*.

eTable 2. Anatomical Therapeutic Chemical (ATC) codes for exposure and covariates

Medications	ATC codes
Metformin	A10BA02, A10BD02, A10BD03, A10BD05, A10BD07, A10BD08, A10BD10, A10BD11, A10BD13, A10BD14, A10BD15, A10BD16, A10BD17, A10BD18, A10BD20, A10BD22, A10BD23
Nonsteroidal anti-inflammatory drugs or aspirin	M01A, N02BA, B01AC06
Statins	C10AA, C10B

© 2026 Xie S et al. *JAMA Network Open*.

eTable 3. International Classification of Diseases (ICD) codes for smoking-related diagnoses

Classification	Codes
ICD-10	*All the Nordic countries:* Z72.0, F17.2, J40-J44, J47
ICD-9	*Finland:* 3051A, 490, 491, 492, 494, 496
	Sweden: 305B, 490, 491, 478X, 292A, 292B, 292C, 294A, V15W, V15X, 779W, 779X, V13W, V13X, V65E, 492, 496, 494
ICD-8	*Denmark:* 49009, 49100, 49101, 49102, 49104, 49108, 49109, 49200, 49201, 49208, 49209

© 2026 Xie S et al. *JAMA Network Open*.

eTable 4. International Classification of Diseases (ICD) codes for alcohol-related diagnoses

Classification	Codes
ICD-10	*All the Nordic countries:* F10, G31.2, G62.1, G72.1, I42.6, K29.2, K70, K86.0, Z71.4, T51.0, T51.9
ICD-9	*Finland:* 291, 3050A, 4255A, 5353A, 5711A, 5770D-F, 5771C-D 303, 9800A
	Sweden: 291, 303, V79B, 305A, 425F, 535D, 571A-571D, 577B, E860, E862
ICD-8	*Denmark:* 29119, 29139, 29199, 30320, 30328, 30329, 30391, 57109, 57110, 57710, 30399
	Sweden: 291, 303, E8699, 98000

© 2026 Xie S et al. *JAMA Network Open*.
