# Supplementary material for: Metformin Use and Development of Esophageal Squamous Cell Carcinoma
Source: JAMA Netw Open. 2026 Mar 16;9(3):e262027. doi: 10.1001/jamanetworkopen.2026.2027 (PMC12993697; doi:10.1001/jamanetworkopen.2026.2027)
Supplement: Supplement 2. — Data sharing statement [file jamanetwopen-e262027-s002.pdf]

Data Sharing Statement

Xie. Metformin Use and Development of Esophageal Squamous Cell Carcinoma. *JAMA Netw Open*. Published March 16, 2026. doi:10.1001/jamanetworkopen.2026.2027

Data
Data available: No
